# Unintended Side Effects of Transformation Are Very Rare in *Cryptococcus neoformans*

**DOI:** 10.1534/g3.117.300357

**Published:** 2018-01-05

**Authors:** Ryan Z. Friedman, Stacey R. Gish, Holly Brown, Lindsey Brier, Nicole Howard, Tamara L. Doering, Michael R. Brent

**Affiliations:** * Center for Genome Sciences and Systems Biology, Washington University School of Medicine, Saint Louis, Missouri 63110; ‡ Department of Molecular Microbiology, Washington University School of Medicine, Saint Louis, Missouri 63110; § Department of Genetics, Washington University School of Medicine, Saint Louis, Missouri 63110; † Department of Computer Science and Engineering, Washington University, Saint Louis, Missouri 63130

**Keywords:** Reverse genetics, gene deletion, yeasts, genome sequence, *Cryptococcus neoformans* strain KN99

## Abstract

Received wisdom in the field of fungal biology holds that the process of editing a genome by transformation and homologous recombination is inherently mutagenic. However, that belief is based on circumstantial evidence. We provide the first direct measurement of the effects of transformation on a fungal genome by sequencing the genomes of 29 transformants and 30 untransformed controls with high coverage. Contrary to the received wisdom, our results show that transformation of DNA segments flanked by long targeting sequences, followed by homologous recombination and selection for a drug marker, is extremely safe. If a transformation deletes a gene, that may create selective pressure for a few compensatory mutations, but even when we deleted a gene, we found fewer than two point mutations per deletion strain, on average. We also tested these strains for changes in gene expression and found only a few genes that were consistently differentially expressed between the wild type and strains modified by genomic insertion of a drug resistance marker. As part of our report, we provide the assembled genome sequence of the commonly used laboratory strain *Cryptococcus neoformans var. grubii* strain KN99α.

One of the fundamental techniques of molecular biology is editing a genome by integrating a DNA molecule at a specific site. The goal may be to introduce a new sequence, eliminate an existing sequence, or both. This technique is widely used on both a gene-by-gene scale and a genome-wide scale. The first comprehensive, targeted gene knockout collection was made by replacing 96% of open reading frames in the genome of *Saccharomyces cerevisiae* with a drug resistance cassette (Winzeler *et al.* 1999; Giaever *et al.* 2002; Giaever and Nislow 2014). Since then, deletion collections have been made for *Schizosaccharomyces pombe* (Kim *et al.* 2010; Spirek *et al.* 2010) and *Neurospora crassa* (Dunlap *et al.* 2007; Roche *et al.* 2014), and a collection for *Cryptococcus neoformans var. grubii* is currently under construction. In these organisms, the first step in genome editing is transformation with linear DNA containing a selectable drug resistance marker and targeting sequences on both ends, which are homologous to the flanking regions of the genome sequence to be replaced. Homologous recombination swaps the exogenous DNA for the target DNA, enabling the recombinant cells to survive selection on a drug that is lethal to cells lacking the resistance marker.

Despite the widespread use of transformation, it has long been thought that this process may lead to unwanted, collateral mutations that confound studies of the intended mutations (Shortle *et al.* 1984; Giaever *et al.* 2002; Johnston *et al.* 2002; Scherens and Goffeau 2004). However, the evidence cited in support this claim is circumstantial (see *Discussion*). The worry about transformation being mutagenic has been even greater among researchers making targeted deletions in *C. neoformans*, an opportunistic, pathogenic fungus that is estimated to cause several hundred thousand deaths per year (Rajasingham *et al.* 2017). Transformation of this yeast, which is surrounded by a thick cell wall and a polysaccharide capsule (Srikanta *et al.* 2014), requires biolistic DNA delivery. In this process, DNA-coated gold beads are fired at the cells at high velocity, penetrating both the capsule and the cell wall (Armaleo *et al.* 1990). The violence of biolistic transformation has led to concerns that it may physically damage chromosomes, leading to abnormal karyotypes.

It is important to distinguish between (a) collateral damage from the transformation process itself and (b) mutations that arise after transformation and are selected for because they compensate for a reduction in fitness due to deletion of a gene. There is evidence to support the latter (see *Discussion*), but the former has never been studied directly.

In this paper, we present the first analysis and characterization of the collateral mutations from genetic transformation—and any potential impact on gene expression—using *C. neoformans var. grubii*, strain KN99α (haploid cells). We carried out 23 independent biolistic transformations on *Cryptococcus*, in each of which a drug marker was inserted in an apparently neutral site (J. Lodge, personal communication) on chromosome 11 (Figure 1, left). We characterized these transformants for virulence-related phenotypes that are frequently studied in *Cryptococcus*, carried out RNA sequencing (RNA-seq) on a subset of transformants to identify potential effects on gene expression, and sequenced the 23 genomes to high coverage. As a control for preexisting variation in the freezer stock, we also sequenced 30 nontransformed cultures, each inoculated by selecting single colonies after streaking out of the same freezer vial (Figure 1, right). For comparison, we also sequenced the genomes of six strains in which the same drug markers had been used to delete genes (Figure 1, center).

**Figure 1:**
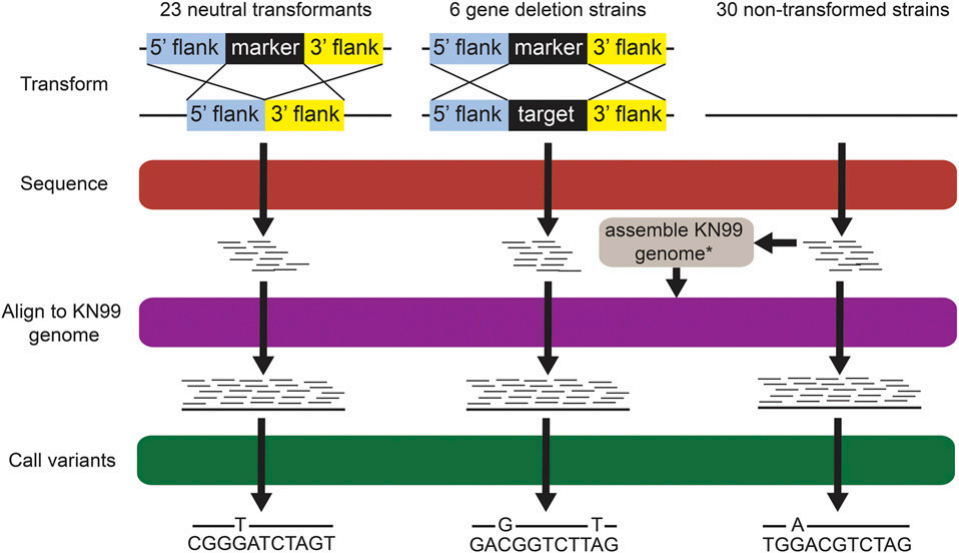
Overview of the study design and procedures. The KN99α genome assembly was guided by the H99α genome sequence, as described in Supplemental Methods in File S2.

The reference genome sequence for *C. neoformans var. grubii* was obtained from laboratory strain H99α (Loftus *et al.* 2005; Janbon *et al.* 2014). Since then, another laboratory strain, KN99α (Nielsen *et al.* 2003), has become increasingly popular, because it is nearly congenic to H99α but exists in both mating types and is competent to mate. As a fringe benefit of our study of transformation, we obtained the genome sequence of strain KN99α, which has not been previously published. We have deposited that sequence, along with annotations transferred from H99α, in public databases, and we provide a brief description of the differences between the KN99α and H99α genomes.

## Materials and Methods

### Cell growth, transformation, and phenotypic assays

Unless indicated otherwise, cells were grown on yeast peptone dextrose (YPD) at 30°, with shaking (∼230 rpm) for liquid cultures. Biolistic transformation was performed as previously described (Toffaletti *et al.* 1993) for genomic integration of marker cassettes (“neutral transformations”) and for gene deletion [as in Maier *et al.* (2015)]. For phenotypic assays, 5 aliquots of 10-fold serial dilutions were spotted and incubated at 30 or 37° on medium containing various stressors. (Details of these and other procedures are provided in Supplemental Material, Supplemental Methods in File S2.)

### RNA-seq and gene expression analysis

Strain growth, RNA isolation, and RNA-seq library preparation were performed as in Gish *et al.* (2016) in biological triplicate, and libraries were sequenced on an Illumina HiSeq 2500. Genes were analyzed for differential expression using DESeq 1.24.0 (Anders and Huber 2010); a gene was considered consistently differentially expressed in a strain if its adjusted *P*-value was <0.01 in all triplicate sets and its expression level changed by two-fold or greater in at least one of the triplicate sets compared with the control.

### Genome sequencing

Genomic DNA was isolated and libraries prepared as detailed in the Supplemental Methods in File S2, with sequencing on an Illumina HiSeq 2500 to obtain paired-end 101 bp reads.

### Genome read alignment

Genome sequencing reads were aligned to the reference genome sequence using both Bowtie 2.2.5 (Langmead and Salzberg 2012) and BWA-MEM 0.7.12 (Li 2013) with default parameters. PCR duplicates were removed using the rmdup command from SAMtools 1.3 (Li *et al.* 2009).

### Copy number variant calling

Copy number variants (CNVs) were called with CNVnator 0.3.2 (Abyzov *et al.* 2011) using default parameters, based on both Bowtie2 and BWA-MEM alignments. Each CNV is assigned a mean depth of coverage relative to the genome-wide depth of coverage. CNVs were considered duplications if the read depth was at least 1.9 times the genome-wide average for the strain. CNVs were considered deletions if the normalized depth was no more than 0.25 times the genome-wide average. All CNVs that overlapped with repeats identified with RepeatMasker 4.0.5 (Smit *et al.* 2013) using default parameters were discarded.

### Structural variant calling

Reads from BWA-MEM alignments were processed through SAMBLASTER 0.1.24 (Faust and Hall 2014) to separate discordant read pairs and split reads from all other reads. The split and discordant reads were input to LUMPY (Layer *et al.* 2014) with default parameters to call structural variants.

### SNP and indel calling and analysis

After alignment, the nonsplit, concordant read pairs were filtered with a mapping quality threshold of 1 for BWA-MEM and 42 for Bowtie2. These thresholds eliminate reads that align equally well to multiple locations in the reference sequence, such as in low complexity and/or repetitive regions. Next, Freebayes 1.1.0 (Garrison and Marth 2012) was used with the parameter -p 1 to call single-nucleotide polymorphisms (SNPs) and indels on Bowtie2 and BWA-MEM alignments. Only high-quality polymorphisms (*P* < 10^-7^) were kept. See Supplemental Methods in File S2 for additional details.

### KN99α reference genome construction

Reads from the 30 nontransformed strains were used to produce the KN99α genome sequence as described in Figure S1 in File S1. This sequence is archived as National Center for Biotechnology Information (NCBI) BioSample SAMN06704791.

### Data availability

All genome sequence reads have been deposited in the NCBI Sequence Read Archive under accessions SRS2184920, SRS2220286, SRS2220449, SRS2220125, SRS2220451, SRS2220454, SRS2220455, SRS2220462, and SRS2220463. The KN99α genome sequences and annotations we used are archived at NCBI (BioProject accession PRJNA384617, assembly and annotation accession ASM221672v1, and BioSample accession SAMN06704791). All RNA-seq data has been deposited in the NCBI GEO database under accession GSE104198.

## Results

### Transformation

All transformed strains received cassettes conferring resistance to either geneticin (G418) (Hua *et al.* 2000) or nourseothricin (NAT) (McDade and Cox 2001), which included 1000 bp of the sequence 5′ and 3′ of the genomic region to be replaced (Figure 1). Cells were transformed using a split marker approach (Fu *et al.* 2006; Kim *et al.* 2009), in which the cassette is split across two segments of linear DNA that overlap by 500 bp. Resistance is conferred only when homologous recombination fuses the two overlapping segments and integrates them into the genome, replacing everything between the targeted flanking regions. The linear DNA was coated onto 0.6 µm gold beads and delivered to a concentrated patch of cryptococcal cells on rich YPD medium using a Bio-Rad PDS-1000 high-velocity biolistic particle delivery vacuum system (Toffaletti *et al.* 1993). In 23 of the strains, the cassette was targeted to the same apparently neutral site in the genome (neutral transformants; Figure 1, left). In another six strains, the cassette was targeted to replace the complete open reading frame of a transcriptional regulator (Figure 1, center). These strains were selected from among a much larger set of regulator deletion strains, because RNA-seq data (Maier *et al.* 2015) revealed that their drug resistance cassettes (three G418, three NAT) had unusually high or low expression, suggesting the possibility of multiple marker integration events or partial marker integration. After biolistic transformation, the transformed cells were incubated overnight at 30°, transferred to fresh YPD plates containing the appropriate drug, and grown for a further 48–72 hr; single colonies were then restreaked onto the same medium for growth and analysis. PCR- and sequence-validated strains were frozen down and used for the remainder of this study.

### Phenotypes

The 23 neutrally transformed strains were tested for growth at 37° in the presence of high-salt (1.2 M KCl or NaCl), cell wall or membrane stressors (0.5% Congo red, 0.01% sodium dodecyl sulfate, 0.2% calcofluor white, or 6% ethanol), or host-like oxidative or nitrosative stress inducers (1 mM H_2_O_2_ or 0.5 mM NaNO_2_). They were also tested for the ability to produce melanin when grown on medium containing L-DOPA, a characteristic associated with cryptococcal virulence. Finally, the width of their capsules was measured. No abnormal phenotypes were observed (data not shown).

### Genome sequencing and analysis

We sequenced the genomes of the 23 neutral transformants, six regulator deletion strains, and 30 nontransformed control strains. To obtain the control strains, the same freezer vial was sampled 30 times onto independent YPD plates. After restreaking, a single colony was grown and treated in a way that mimicked the transformation process, except that biolistic transformation was skipped and the drug was omitted from subsequent growth on YPD plates. We extracted genomic DNA from all 59 strains, prepared Illumina sequencing libraries, and obtained paired-end 101 bp reads by Illumina sequencing.

### Sequencing and assembly of C. neoformans var. grubii strain KN99α

The reads from the 30 wild-type control strains were aligned to the H99α reference genome sequence and variants were called. Variants that occurred in all or all-but-one of the nontransformed strains were used to construct a reference genome sequence for KN99α. Attempts to assemble the small fraction of reads that could not be mapped to the H99 reference did not reveal any significant KN99 contigs that failed to map to H99. After completing an iterative alignment and refinement process (Figure S1 in File S1), the gene annotation from H99α was transferred to KN99α, with adjustments dictated by the new sequence. This revealed a 70-bp displaced duplication within a 3′ untranslated region (UTR) and four large deletions. The deletions affect the 5′ UTRs of two genes and eliminate *SGF29* (CNAG_06392) almost completely (Table S1 in File S1). The loss of *SFG29* in the KN99 lineage was recently observed by another group, which attributed the relatively high virulence of KN99 to it (Arras *et al.* 2017). The other major feature of the KN99α sequence was a 285 kb region (chromosome 11:175,000–460,000) that was highly diverged from H99α. Within the diverged region, there were 4301 substitutions (one per 66 bp, *vs.* one per 153,778 bp in the remainder of the genome) and 369 small insertions or deletions (one per 772 bp, *vs.* one per 331,836 bp in the remainder of the genome). The differences from H99α affected 108 of 115 proteins encoded in the variable region. This variable region can be traced to the original creation of KN99α, in which H99α was crossed with a strain derived from clinical isolate 8–1 and then backcrossed to H99α 10 times (Nielsen *et al.* 2003; Janbon *et al.* 2014). The 285 kb divergent region matches the sequence from strain 8-1 and was retained despite the 10 backcrosses to H99α.

### Variants in KN99α

To identify variants within our freezer stock of wild-type KN99α, we aligned the reads from the 30 nontransformed strains to the new KN99α genome sequence and filtered out dubious variants, as described in Supplemental Methods in File S2. At the end of this process, only seven variant sites remained. Six of these fell in the diverged region of chromosome 11 (Table S3 in File S1). At four of these sites there was only a single alternate (*i.e.*, nonreference) allele. At the remaining three sites all 30 genomes differed from H99α, but there was variation within the KN99α freezer population itself (Table S3 in File S1, column “Alt.”). Notably, in each genome a significant fraction of reads supported each allele at the six variant sites on chromosome 11 (Table 1, column “MF”), suggesting variation among the cells in each culture. These appear to be due to genuine ambiguity in the detailed alignments of the reads, as most have secondary alignments that are very near to the primary in genome coordinates and in alignment score. One variant in the 30 nontransformed strains occurred on a different chromosome, was supported by 95% of the aligned reads, and occurred in only one strain (Table 1, “30 Nontransformed”). This appears to be a genuine polymorphism.

**Table 1:** Variant alleles found in 30 control strains (top), 23 neutrally transformed strains (middle), and six gene deletion strains (bottom)

30 Nontransformed										
Chr	Pos	Reference	Alternate	AC	Strain	AO	RO	MF (%)	Gene	Effect
4	574814	TAA	TAAA	1	WT	54	3	95	05155*^a^*	Promoter

23 Neutrally Transformed										
Chr	Pos	Reference	Alternate	AC	Strain	AO	RO	MF (%)	Gene	Effect
2	22453	G(T)_11_C	G(T)_12_C	1	G418_20	10	0	100	06794*^b^*	Intron
2	303154	T	G	1	NAT_40	29	0	100	*STE6**^c^*	Silent
3	422414	C	T	1	NAT_6	48	0	100	*LIV10**^d^*	E508K
14	79613	G	C	2	G418_28	36	0	99	*CAT2**^e^*	Promoter
					G418_47					

Six Gene Deletion Strains										
Chr	Pos	Reference	Alternate	AC	Strain	AO	RO	MF (%)	Gene	Effect
1	1524172	A	C	1	*pkr1∆*	49	0	100	00593*^f^*	Silent
1	2183532	G	T	1	*yrm103∆*	24	0	100	00823*^g^*	Silent
2	1492740	A	G	1	*ssn801∆*	25	0	100	04048*^h^*	V471A
5	410958	G(A)_10_T	G(A)_9_T	1	*pkr1∆*	33	1	97	01397*^i^*	3′ UTR
6	91956	A	G	1	*ssn801∆*	28	1	97	02524*^j^*	Silent
6	1082445	C	T	1	*yrm103∆*	41	2	95	02146*^k^*	T236I
7	1162733	G	A	1	*ssn801∆*	21	0	100	05900*^l^*	S29L
8	531945	G	C	1	*ccd6∆*	56	0	100	03269*^m^*	P58R
9	1050856	G	C	1	*yrm103∆*	31	0	100	*VPS4**^n^*	P110A
14	837564	AG(TCG)_4_	AG(TCG)_5_	1	*ssn801∆*	13	0	100	05636*^o^*	5′ UTR
1	1231133	TCA	GAG	6	All 6	5	0	98	07362*^p^*	E68S
7	1278590	ATT	ATTT	6	All 6	27	2	91	N/A	Intergenic

Chr, chromosome on which the variant occurs; Pos, coordinate of the variant in the KN99α reference genome sequence; Reference, reference allele; Alternate, alternate allele; AC, “alternate count,” the number of strains with the alternate allele; Strain, the strain or strains in which the alternate allele is found; AO, “alternate observations,” the mean number of reads supporting the alternate allele in strains with the alternate allele; RO, “reference observations,” the mean number of reads supporting the reference allele in strains with the alternate allele; MF, “mean fraction,” the mean fraction of reads supporting the alternate allele in strains with the alternate allele; Gene, the name or KN99 gene identifier (CKF44 number) of the gene affected by the variants; Effect, the effect of the variant on the protein encoded by the indicated gene or location in the indicated gene.

a Protein-tyrosine-phosphatase.

b Hypothetical protein.

c Pheromone transporter.

d Homolog of S. cerevisiae gene RPP1.

e Catalase.

f Homolog of *S. cerevisiae* gene *AIM18*

g Cadmium ion transporter.

h Brg1-associated factor b.

i Mitochondrial membrane transport.

j Homolog of *S. cerevisiae* gene *YKL111C*.

k Homolog of *S. cerevisiae* gene *ALE1*.

l Glycine-tRNA ligase.

m Aldehyde dehydrogenase.

n ATPase.

o Homolog of *S. cerevisiae* gene *TIF4632*.

p Single-stranded DNA binding protein.

A limitation of this and all following analyses is that, as expected, 4.15% of the H99 genome was not covered by an average of at least five uniquely mapped reads, when averaged across all 30 nontransformed strains. This includes centromeres and other repetitive DNA. Variants found in these areas might be missed by our analysis owing to their repetitive nature.

### Side effects of transformation

**Figure 2:**
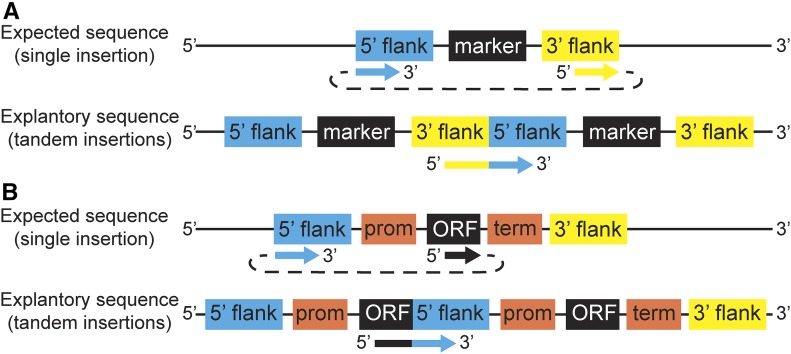
Evidence from split read alignments for tandem insertions. The top part of each panel shows the expected insertion and the aligned, observed sequence (colored arrows connected by a dotted line indicate the sequence of the read). The bottom part of each panel explains this observation. (A) Multiple insertions of the complete marker cassette with targeting flanks. (B) Partial sequences lacking the terminator and 3′ targeting flank.

After removing all of the variants found in the nontransformed control strains, eight high-confidence variant sites remained in the 23 neutrally transformed strains. Four of these fell within one of the targeting flanks of the inserted DNA (Table S2 in File S1, top). These may simply reflect PCR errors that occurred prior to transformation and cannot be considered side effects of transformation. The other four (Table 1, “23 Neutrally Transformed”) appear to be genuine mutations. In three cases, the alternate allele occurred in only one strain, and the fourth occurred in only two strains (Table 1, column “AC”). Furthermore, these alternate alleles were supported by 99–100% of reads (Table 1, column “MF”). In three of the four, read depth at the variant sites was typical of the genome-wide coverage (Table 1, column “AO”). These results suggest that side-effect mutations may occur, although the frequency of mutations in the transformants (4 out 23) was not different from their frequency in the control strains (1 out 30) at a statistical significance threshold of 0.05 (Fisher’s exact *P* < 0.14). Thus, transformation-induced mutations, if they exist at all, are strikingly rare: we observed an average of only 0.22 mutations per neutrally transformed strain. We also observed one partial multiple insertion of the drug marker in these strains (Figure 2B).

### Side effects of transformation with gene deletion

For the six strains in which a gene was deleted, the situation was different from both the nontransformed and neutrally transformed strains. Thirteen variant sites were identified across these strains. One of these fell within the targeting flank of the linear DNA that was transformed into the cells (Table S2 in File S1, bottom row). The other 12 fell in genomic regions that were apparently unrelated to the region targeted—11 of them were on different chromosomes from the targeted gene. These 12 had good coverage (Table 1, column “AO”), and nearly 100% of reads supported the alternate alleles (Table 1, column “MF”). Two of them occurred in all six gene deletion strains and likely represent drift in the freezer stock resulting from laboratory passage, as discussed below. The other 10 occurred in only one strain each (Table 1, column “AC”), yielding an average of 1.67 mutations per gene deletion strain, notably higher than the rate in neutrally transformed strains.

We also found evidence for multiple tandem insertions of the marker in three of the six gene deletion strains and one of the 23 neutrally transformed strains. The higher frequency in the gene deletion strains is not surprising—these strains were selected for sequencing because they expressed the drug marker at very high or very low levels. These multiple insertions were initially identified by the LUMPY software (Layer *et al.* 2014) on the basis of split reads (Figure 2) and are independently and strongly supported by read depths that are many times the genome-wide average. Two of the multiple insertion strains express the marker at a very high level and one expresses it at a very low level. One possible explanation for the latter is that the marker could have inserted into a poorly expressed region of the genome, so that only strains with multiple copies could express it at a high enough level to survive the drug. This hypothesis is supported by the observation that the gene targeted for deletion had very low expression in our RNA-seq data (Maier *et al.*, 2015) .

### Variants fixed by a bottleneck during laboratory passage

Two point mutations occurred in all six gene deletion strains. These seemed unlikely to represent rare side effects of transformation or compensation for loss of a deleted gene, since the six strains carried deletions of six different genes. We noticed that all six deletion strains were constructed in July 2010, whereas our KN99α reference sequence was based on cultures inoculated from cells streaked from a frozen stock in March 2016. This led us to hypothesize that the “alternate” alleles were actually the major alleles prior to a bottleneck event that occurred between these dates, potentially when the freezer stock was depleted and a new stock was grown from a single colony of the old stock in late 2011. To investigate the possibility of a bottleneck fixing random mutations, we called variants in transcribed regions using RNA-seq reads (Maier *et al.* 2015). We identified four other SNPs with allele frequencies of 5–11% in 84 strains cultured in 2010 and 2011 and frequencies of 93–100% in 104 strains cultured since January 2012. Although neither of the two SNPs listed in Table 1 was in an expressed region, the existence of other SNPs that went rapidly to fixation at the same time supports the theory that a bottleneck event in late 2011 led to fixation of the current KN99α sequence, eliminating what was the major allele at the time in the freezer stock used to construct the six gene deletion strains.

### Gene expression analysis by RNA-seq

To determine whether any mutations induced by transformation affected gene expression, we carried out RNA-seq on three strains that were neutrally transformed with the G418 drug resistance cassette, three that were neutrally transformed with the NAT resistance cassette, and one untransformed wild type, each in biological triplicate. We then focused on genes that were differentially expressed by at least two-fold (adjusted *P*-value <0.05), leaving us with between one and 13 differentially expressed genes for five of the strains and one outlier strain (NAT_6) with 65. Five genes (CKF44_00549, CKF44_01093, CKF44_01680, CKF44_02711, CKF44_06402) were differentially expressed in all three NAT transformants, of which one (CKF44_00549) was also differentially expressed in all three G418 transformants. Apart from CKF44_01680, which is homologous to a methylcrotonoyl-coenzyme A carboxylase, these five consistently differentially expressed genes are all hypothetical conserved proteins with no gene ontology annotations and no known domains. These consistently differentially expressed genes were not clustered in one region of the genome, and none was near the marker insertion site. Apart from these, each strain contained only a few differentially expressed genes, except for NAT_6 (discussed below).

## Discussion

After analyzing genomic sequences from 23 strains in which a drug marker had been inserted in an apparently neutral location and 30 nontransformed control strains, we found a very low rate of collateral mutations resulting from the transformation process. All 23 transformations together resulted in only four point mutations, of which one changed an amino acid, and one case of multiple tandem insertions of the drug marker. Furthermore, none of the 23 transformants showed any abnormal stress resistance phenotypes.

Our findings stand in striking contrast to the received wisdom in the field, which is that transformation is inherently mutagenic. However, the only published evidence supporting this belief comes from the construction of the yeast knockout (YKO) collection. The YKO collection was constructed by transforming a drug marker cassette into diploid cells, in order to replace one copy of the target gene. The diploids were then sporulated to produce four haploid spores, two of which carry the drug resistance cassette. Only ∼25% of the gene deletions caused slow growth directly—that is, with 100% linkage to the drug resistance trait. However, an estimated 6.5% of the transformed diploids contained a mutation that caused a growth defect that was unlinked to the drug resistance cassette. These growth defects were assumed to result from collateral mutations that affect gene function. Since only 25% of such mutations would be expected to yield growth defects, it was estimated that the total collateral mutation rate was 6.5% / 25% = 26% of diploids (Giaever *et al.* 2002).

There are several possible explanations for the discrepancy between our sequencing-based finding and the inference made from the YKO collection. First, we transformed haploid strains, so any mutants that displayed slow growth in haploids would be unlikely to be picked after growth on plates with selective media. However, we would still expect to see the remaining three quarters of the collateral mutations (75 * 26% = 19.5%), which do not affect growth in rich medium, in our genome sequences. The fact that we did not may result from several differences in the two transformation protocols. The YKO protocol involves permeabilizing cells with lithium acetate, whereas we used biolistic transformation. It has been thought that the biolistic process might damage chromosomes, but we saw no evidence of structural variants or CNVs in any of the 29 neutrally transformed strains. It is possible that the lithium acetate protocol is more mutagenic than biolistic transformation. Another important difference is that our drug cassette was split between two linear DNA segments with overlapping sequences to support homologous recombination between them (“split marker transformation”) (Kim *et al.* 2009; Fu *et al.* 2006), making off-target insertions that yield drug-resistant transformants less likely. Finally, each of the DNA pieces we used for transformation contained 1000 bases of targeting homology, whereas the YKO protocol uses only 45–80 bp of targeting homology on each end. The shorter homologous segments, combined with the transformation of an intact marker cassette, could lead to more secondary, off-target insertions. Anecdotal evidence supporting that idea comes from the recollection that, during construction of the YKO collection, a significant number of drug-resistant transformants retained the gene targeted for replacement (L. Riles, personal communication).

Differences between the DNA repair mechanisms of *S. cerevisiae* and those of *C. neoformans* could, in principle, also contribute to a higher rate of off-target insertions in *S. cerevisiae*. DNA repair mechanisms have not been studied in detail in *C. neoformans*, although it has been observed that *Cryptococcus* has a lower homologous recombination rate than *S. cerevisiae* (Davidson *et al.* 2000; Chun and Madhani 2010) and that this can be mitigated by deleting genes required for nonhomologous end joining (Goins *et al.* 2006).

The one amino acid-changing mutation found in the 23 neutrally transformed strains occurred in *LIV10*, a homolog of *S. cerevisiae RPP1*, which encodes an RNase subunit involved in processing of precursor rRNA and tRNA as well as nuclear RNA turnover. This may explain why the strain bearing this mutation (NAT_6) expressed 65 genes at significantly different levels compared with wild-type cells, many more than the other transformants subjected to RNA-seq. Specifically, the differential expression of many of these genes may be due to changes in RNA maturation or degradation.

It is important to distinguish between mutagenicity of the transformation process, for which we found no evidence, and selection for naturally occurring variants that compensate for the loss of a targeted gene. In the six gene-deletion strains we sequenced, we did see a greater mutation rate than in the neutrally transformed strains. We found an average of 1.67 collateral point mutations and 0.5 cases of multiple tandem marker insertion per transformation. This is substantially higher than the rate found for neutral transformations, but still low in an absolute sense. The tandem marker insertions are certainly not representative of all gene deletion strains, since these strains were selected from a much larger collection of deletion strains because of their extreme marker expression in RNA-seq data. Of the collateral mutations, only four (0.67 per gene deletion strain) affected amino acids and none occurred in genes with obvious relationships to the deleted gene.

The greater number of variant alleles in gene deletion strains is consistent with recent evidence for selection of natural variants after transformation, owing to the loss of a deleted gene (Teng *et al.* 2013). After transformation and selection of a clonal colony, yeast cells are typically grown for ∼30 generations before they are stored, during which one would expect ∼10^7^ mutation events (Teng *et al.* 2013). This would provide ample variation, some of which might be under positive selection in the absence of the deleted gene. Teng *et al.* found that different individual cells within supposedly homogeneous strains of the YKO collection had different phenotypes in at least one of two assays they performed. Of the strains they tested, 72% displayed heritable diversity in these phenotypes that was stable over long periods of time. They concluded that deletion of almost any gene can cause selection for variants that arise naturally during cell culture. These findings suggest that we should see variants supported by 20–80% of reads in at least 70% of our six gene deletion strains. We did not observe such variants, but six deletion strains is a very small sample. To further investigate this, we analyzed alignments of 378 RNA-seq expression profiles (including single and double deletions of >50 genes) for variants showing the pattern that would be predicted by the finding of Teng *et al.* that individual YKO strains are phenotypically diverse. Specifically, we looked for variants covered by at least 10 reads in a sample, including at least five reads supporting the variant, with at least 20% of reads supporting the reference allele and 20% supporting the variant. We only found eight such variants among the 378 samples analyzed. Seven of the eight variants occurred in >1 sample, although they did not occur in multiple samples of the same genotype as would be expected if they had been selected for by the loss of the deleted gene. In summary, we did not see the evidence of diversity within cultures that would be expected based on the findings of Teng *et al.*

Our finding of 1.67 point mutations per gene deletion strain is broadly consistent with recent sequencing-based studies in *S. cerevisiae*. A study of deletion mutants with slow growth phenotypes that showed large improvements after 400 generations of laboratory passage found an average of six point mutations and 0.5 small indels per strain (Szamecz *et al.* 2014). The slightly larger number is not surprising given the selection of the strains for initial gene knockouts that were deleterious and the much larger number of generations of laboratory passage. A study of suppressor mutants found an average of eight mutations that the authors interpreted as being noncompensatory, in addition to the compensatory mutation (van Leeuwen *et al.* 2016). Many of these mutations were in genes that are known to be frequently mutated after laboratory passage that ends in nutrient-limited stationary phase, likely because the mutations delay the slowing of growth in response to nutrient limitation (Comyn *et al.* 2017). Many of the strains sequenced were selected because they were suspected to have an off-target mutation, and the rest were selected based on a slow growth phenotype linked to a marker gene. Thus, they were not a random sample of deletion mutants. A third study found one to four private SNPs per strain in six gene deletion strains and 0.8–1.6 in 10 strains transformed with a barcode-marker construct that did not delete a gene (Jaffe *et al.* 2017).

Although widespread aneuploidy in the YKO strains has been reported (Hughes *et al.* 2000), we found no evidence for aneuploidy in our six deletion mutants or 23 neutrally transformed strains. We also searched for evidence of aneuploidy in our RNA-seq data consisting of 233 wild-type samples, 139 gene deletion samples, and six overexpression samples. We did not see any sign of aneuploidy on a whole-chromosome scale, although it is possible that there were duplications of <500 kb. One possible explanation for the difference between our findings and those of Hughes *et al.* is based on their observation that, in yeast, the duplicated region often contained a gene that was highly homologous to the deleted gene. It could be that *Cryptococcus* has fewer such highly homologous gene pairs, since *S. cerevisiae* underwent a whole-genome duplication within the last 100 My.

In conclusion, we have found biolistic transformation, using split markers flanked by 1 kb of targeting homology, to be a safe process with few unintended consequences. Thus, investigators should feel confident in constructing mutant strains by this process and even using it repeatedly to effect multiple changes in the same strain. In contrast to the transformation process itself, gene deletion may select for a few spontaneous point mutations. However, such mutations, even when they change a single amino acid, are much less likely to cause a visible phenotype than deleting an entire gene, so any observed phenotypes are likely attributable to the intended deletion; this can be confirmed by complementation.

We also found that regrowing freezer stocks from a single colony can cause bottlenecks that fix variants within the freezer population. Each bottleneck could fix multiple variants, causing increasing divergence from the reference isolate over multiple bottleneck events. We recommend that investigators keep a single, master population in the freezer that has undergone as few passages from the reference isolate as possible. Contrary to standard practice, we recommend that when expansion of this master population is needed, it should be done without selecting a single colony.

## Supplementary Material

Supplemental material is available online at www.g3journal.org/lookup/suppl/doi:10.1534/g3.117.300357/-/DC1.
