# Supplementary material for: Unintended Side Effects of Transformation Are Very Rare in Cryptococcus neoformans
Source: G3 (Bethesda). 2018 Jan 5;8(3):815–22. doi: 10.1534/g3.117.300357 (PMC5844303; doi:10.1534/g3.117.300357)
Supplement: Supplementary file 1 [file 815FileS1.docx]

SUPPLEMENTAL FIGURES AND TABLES

Fig. S1: Construction of the KN99 genome. Sequencing reads from 30 independent cultures of wild type KN99 were aligned to the H99 genome; structural variants (SVs) and copy number variants (CNVs) were substitutions into the H99 genome sequence, yielding the intermediate that we call KN99 version 0. The reads were then re-aligned to KN99.0 using BWA-MEM (mapping quality (MQ) threshold 1) and Bowtie2 (MQ threshold 42). Small base changes, insertions, or deletions were identified by running Freebayes 1.1.0 {Garrison & Marth, 2012} on the alignments produced by each aligner (VCF quality (QUAL) threshold 70). Starting with the variants called from Bowtie2 alignments, we filtered out variants that were not supported by at least five reads (11% of genome-wide average). Many of these poorly supported variants only affected the lengths of mono-nucleotide repeats, which are difficult to determine accurately by Illumina sequencing. We also filtered out variants that did not overlap a variant called from the BWA-MEM alignments, enforcing a degree of consensus. These well supported variants were then substituted into the preliminary KN99 sequence by using the vcf-consensus command from the Perl module of VCFtools 0.1.14 {Danecek et al., 2011} to produce the next iteration. This pipeline went through 8 iterations to make the final sequence, KN99.8. After the fourth iteration of SNP and indel calling, variants were only kept if they were supported by an average of at least five reads.

| **Chr** | **Pos (H99)** | **Size (bp)** | **Description** |
| --- | --- | --- | --- |
| 3 | 2676-2845 | 169 | Intergenic deletion |
| 7 | 833,157-833,450 | 293 | Deletion including 5’ UTRs of CNAG_05771^1^ and CNAG_05772^2^ |
| 11 | 635,489-635,560 | 72 | Displaced duplication between chr11:310,318 and chr11:310,319 |
| 13 | 367,683-368,316 | 733 | Deletion within *SGF29*^3^ |
| 14 | 170,944-171,334 | 390 | Intergenic deletion |
| ^1^Control telomere length, ^2^*ARP8* homolog, ^3^SAGA histone acetyltransferase complex subunit. | | | |

**Table S1:** Copy-number variants (CNVs) and structural variants (SVs) in KN99 relative to the H99 reference genome. The deletion within *SGF29* includes 70% of the coding region, likely rendering the gene non-functional. Chr: chromosome on which the variant occurs. Pos: Coordinate of the variant in the H99 reference genome sequence.

| **Chr** | **Pos** | **Ref** | **Alt** | **AC** | **Strain** | **AO** | **RO** | **MF** |
| --- | --- | --- | --- | --- | --- | --- | --- | --- |
| 1 | 1468278 | T | C | 1 | *pkr1∆* | 457 | 43 | 91.4% |
| 11 | 286935 | T | C | 1 | G418_5 | 13 | 0 | 100% |
| 11 | 287395 | T | A | 1 | NAT_1 | 36 | 0 | 100% |
| 11 | 287587 | T | C | 1 | NAT_1 | 13 | 0 | 100% |
| 11 | 287670 | T(A)_9_G | T(A)_8_G T(A)_10_G | 2 1 | G418_48, NAT_6 G418_B_11 | 17 16 | 1 | 91.7% 84.2% |

**Table S2:** Variants falling within the targeting flanks of the inserted DNA. Chr: chromosome on which the variant occurs. Pos: Coordinate of the variant in the H99 reference genome sequence. Ref: The reference allele. Alt: The alternate allele. AC: “alternate count”, the number of strains with the alternate allele. Strain: The strain or strains in which the alternate allele is found. AO: “alternate observations”, the mean number of reads supporting the alternate allele in strains with the alternate allele. RO: “reference observations”, the mean number of reads supporting the reference allele, in strains bearing the reference allele. MF: “mean fraction”, the mean fraction of reads supporting the alternate allele in strains with the alternate allele.

| **30 Non-Transformed** | | | | | | | | | | |
| --- | --- | --- | --- | --- | --- | --- | --- | --- | --- | --- |
| **Chr** | **Pos** | **Ref** | **Alt** | **AC** | **Strain** | **AO** | **RO** | **MF** | **Gene** | **Effect** |
| 11 | 215771 | CA | CAGTAA | 5 | WT | 21 | 15 | 57% | 01535^1^ | 3' UTR |
| 11 | 290065 | CT | CC | 3 | WT | 9 | 6 | 57% | *BLP4*^2^ | Silent |
| 11 | 290069 | TGCTC | CGCTT | 16 | WT | 9 | 7 | 57% | *BLP4*^2^ | P135S |
| 11 | 214546 | GGATG | GAATC  GGAATC | 22  8 | WT | 16  11 | 0 | 62%  52% | 01534^3^ | 5’ UTR |
| 11 | 215774 | TGA | TAA  TAAGTAA | 22  8 | WT | 19  16 | 0 | 59%  57% | 01535^1^ | 3' UTR |
| 11 | 341449 | TCT | TTT  TT | 15  15 | WT | 6  4 | 0 | 65%  56% | 01577^4^  01578^3^ | Divergent promoters |
| ^1^amino acid permease, ^2^GYP8 homolog, ^3^hypothetical protein, ^4^glutamate dehydrogenase (NADP)  **Table S3**. Sites with significant support for multiple alleles in most of the 30 controls strains. Pos: Coordinate of the variant in the H99α reference genome sequence. Ref: The reference allele. Alt: The alternate allele. AC: “alternate count”, the number of strains with the alternate allele. Strain: The strain or strains in which the alternate allele is found. AO: “alternate observations”, the mean number of reads supporting the alternate allele in strains with the alternate allele. RO: “reference observations”, the mean number of reads supporting the reference allele in strains with the reference allele. MF: ”mean fraction”, the mean fraction of reads supporting the alternate allele in strains with the alternate allele. Gene: The name or CNAG number of the gene affected by the variants. Effect: The effect of the variant on the protein encoded by the indicated gene or location in the indicated gene. | | | | | | | | | | |
