# Supplementary material for: Unintended Side Effects of Transformation Are Very Rare in Cryptococcus neoformans
Source: G3 (Bethesda). 2018 Jan 5;8(3):815–22. doi: 10.1534/g3.117.300357 (PMC5844303; doi:10.1534/g3.117.300357)
Supplement: Supplementary file 2 [file 815FileS2.docx]

SUPPLEMENTARY METHODS

Media, strain growth, and transformation

Unless indicated otherwise, cells were grown on yeast peptone dextrose (YPD) at 30 °C, with shaking (~230 rpm) for liquid cultures. For biolistic transformation, the parental strain KN99α was recovered from a stock vial stored at -80 °C by transferring a small chip of the frozen culture onto a YPD agar plate and streaking for single colonies. A single colony from the plate was used to inoculate a 50-ml culture of YPD, which was grown overnight. Cells were sedimented (4,000 g; 5 min; room temperature (RT)) and resuspended in 3 ml of “regeneration media” (1 M sorbitol, 1 M mannitol, 0.9% yeast nitrogen base, 2.6% glucose, 0.0267% yeast extract, 0.054% bacto-peptone, and 0.133% gelatin). The resulting thick suspension was spread in a ~3 cm diameter disk on a YPD plate and allowed to dry for 4 hours at RT. The spread cells were then subjected to biolistic transformation ([Bio-Rad Biolistic PDS-1000]) as described (Toffaletti *et al.* 1993), incubated overnight, scraped, and plated on the appropriate selective medium (YPD with G418 or nourseothricin). After 2-3 days of growth, single

colonies were selected and restreaked onto the same medium; a fresh single colony was then cultured for DNA isolation and PCR confirmation of sequence insertion at the expected site. Validated transformants were replated on YPD, grown at 30 degrees and a single colony from this plate was inoculated into 3 ml YPD, cultured overnight, sedimented as above, resuspended in 0.8 ml YPD, mixed with an equal volume of 50% glycerol, and stored at -80 degrees.

For the 23 neutral transformations, the G418 or NAT marker cassette was targeted to chr11:287531-287532, in the coordinates of the H99 reference genome sequence (chr11:287563-287564 in the coordinates of our new KN99 genome sequence), a site characterized as a ‘safe haven’ for insertion (J. Lodge, personal communication). The 6 deletion strains (genotypes *ssn801:G418*; *pkr1:G418*; *clr6:G418*; *yrm103:NAT*; *aro8001:NAT*, or *ccd6:NAT*) were constructed as reported in (Maier *et al.*).

Phenotypic assays

For phenotypic assays, cells were grown and collected as above, resuspended at 10^7^/ml in PBS, and 5 μl aliquots of 10-fold serial dilutions were spotted and incubated at 30 or 37 °C on YPD alone; YPD supplemented with the appropriate selective drug, 1.2 M KCl, 1.2 M NaCl, 0.2% calcofluor white, 0.05% Congo red, 0.01% SDS, or 6% ethanol; melanization medium (0.1% L-asparagine, 0.1% glucose,0.3% KH_2_PO_4_, 0.025% MgSO_4_⋅7H_2_O, and 0.01% L-DOPA); or YNB medium (0.67% yeast nitrogen base without amino acids, 2% glucose, 2%agar, 25mM sodium succinate, pH 4.0) supplemented with oxidative or nitrosative stress inducers (0.5 mM H_2_O_2_ or 0.5 mM NaNO_2_).

RNA sequencing and gene expression analysis

Strains were grown for RNA-seq as in (Gish *et al.* 2016). RNA was isolated from liquid cultures with TRIzol RNA Isolation Reagent (Life Technologies) and Poly-A RNA was purified using an mRNA Catcher Plus Plate (Invitrogen). For library preparation, RNA was chemically shattered by incubating (10 min, 75 °C) in the buffer from TURBO DNase I (Invitrogen NC9075048). It was then incubated (5 min, 65 °C) with Random Primers (Invitrogen 48190-011) and dNTP mix (NEB N0447S) at 65 °C for 5 min and the first strand cDNA synthesized using a SSIII kit (Invitrogen 18080-44) in the presence of RNase OUT (Invitrogen 10777-019), incubated as follows: 25 °C for 5 min, 50 °C for 50 min, 70 °C for 15 min, and holding at 4 °C. Second strand synthesis was performed by combining 5X SS buffer (Invitrogen 10812-014), dNTP mix (NEB N0447S), *E. coli* DNA ligase (NEB M02056), DNA Polymerase I (Invitrogen 18010-025), RNase H (Invitrogen 18021-014) and incubating at 16 °C for 2 h. End repair was carried out using the NEB Quick Blunting Kit (E1201S), an “A” base added using Klenow Fragment (3’ to 5’ exo, NEB M0212S) and 1 mM dATP (Invitrogen 18252-015), and Illumina adapters and indices added by PCR. Libraries were sequenced on an Illumina HiSeq 2500.

RNA-seq was done in biological triplicate on three strains for each of the two markers and a non-transformed control, for a total of 9 G418 libraries, 9 NAT libraries, and 3 non-transformed libraries. Reads were aligned to the H99 reference genome using TopHat 2.0.14 (Trapnell *et al.* 2009) and Bowtie 0.12.9 (Langmead *et al.* 2009). Reads that aligned uniquely to the reference sequence were considered for gene expression quantification with HTSeq 0.6.1 (Anders *et al.* 2015). Genes were analyzed for differential expression using DESeq 1.24.0 (Anders and Huber 2010) built under R 3.3.0. One triplicate set (G418_7) appeared to have more differentially expressed genes than expected, so we made new RNA-seq triplicate sets from new cultures for some of the strains. After further sequencing failures and repeats, we had 13 triplicate sets of which 3 appeared to be outliers and were discarded, leaving one triplicate set for each NAT strain, two triplicate sets for G418_7 and G418_10, and three triplicate sets for G418_6. In our combined analysis of these data, a gene was considered consistently differentially expressed in a strain if its adjusted p-value was less than 0.01 in all triplicate sets and its expression level changed by two-fold or greater in at least one of the triplicate sets when compared to the control.

Genome sequencing

For gDNA isolation, cells from overnight cultures were harvested as above, resuspended in 0.5 ml extraction buffer (50 mM Tris-HCl, pH 8.0, 20 mM EDTA, and 1% SDS), and subjected to three 3-minute rounds of bead beating with an equal volume of silica-zirconia beads, alternating with 1 min incubation at 4 °C. The sample was then heated for 10 min at 70 °C, extracted with phenol:chloroform, ethanol precipitated, and the recovered genomic DNA sonicated to an average size of 175 bp. The fragments were blunt ended, and modified by addition of an “A” base to the 3’ ends and ligation of Illumina sequencing adapters at both ends. The ligated fragments underwent amplification for 8 cycles incorporating a unique indexing sequence tag. The resulting libraries were sequenced on an Illumina HiSeq-2500 to obtain paired end 101 bp reads.

Genome read alignment

Genome sequencing reads were aligned to the reference genome sequence by using both Bowtie 2.2.5 (Langmead and Salzberg 2012) and BWA-MEM 0.7.12 (Li) with default parameters. PCR duplicates were removed using the rmdup command from SAMtools 1.3 (Li *et al.* 2009).

Copy-number variant calling

Copy-number variants (CNVs) were called with CNVnator 0.3.2 (Abyzov *et al.* 2011) using default parameters, based on both Bowtie2 and BWA-MEM alignments. Each CNV is assigned a mean depth of coverage relative to the genome-wide depth of coverage. CNVs were considered duplications if the read depth was at least 1.9 times the genome-wide average for the strain. CNVs were considered deletions if the normalized depth was no more than 0.25 times the genome-wide average. All CNVs that overlapped with repeats identified with RepeatMasker 4.0.5 (Smit *et al.* 2013) using default parameters were discarded.

Structural variant calling

Reads from BWA-MEM alignments were processed through SAMBLASTER 0.1.24 (Faust and Hall 2014) to separate discordant read pairs and split reads from all other reads. The split and discordant reads were input to LUMPY (Layer *et al.* 2014) with default parameters to call structural variants.

SNP and indel calling and analysis

After alignment, the non-split, concordant read pairs were filtered with a mapping quality (MQ) threshold of 1 for BWA-MEM and 42 for Bowtie2. These thresholds eliminate reads that align equally well to multiple locations in the reference sequence, such as in low complexity and/or repetitious regions.

Freebayes 1.1.0 (Garrison and Marth 2012) was used with the parameter -p 1 to call SNPs and indels on Bowtie2 and BWA-MEM alignments. Only high-quality polymorphisms (*P* < 10^-7^) were kept. Called variants based on Bowtie2 alignments were filtered to include only those that overlap at least partially with a BWA-MEM variant. Subsequent analyses were only based on variants called with Bowtie2 alignments. Polymorphisms were considered fixed in KN99α if they occurred in all strains in which an allele was called, or all but one strain. Polymorphisms were considered segregating if at least two strains were assigned the polymorphism and at least two strains were assigned the reference allele. Variants that cause amino acid changes were identified using SnpEff 4.1 (Cingolani *et al.* 2012) The genome sequences and annotations we used are archived with Genbank accession PRJNA177334 (<https://www.ncbi.nlm.nih.gov/genome?linkname=nuccore_genome>).

KN99α reference genome construction

Reads from the 30 non-transformed strains were used to produce the KN99α as described in Supplemental Figure S1. This sequence is archived as NCBI BioSample SAMN06704791.

Data availability

All genome sequence reads have been deposited in the NCBI Sequence Read Archive under accessions SRS2184920, SRS2220286, ﻿SRS2220449, SRS2220125, SRS2220451, ﻿SRS2220454, ﻿SRS2220455, SRS2220462, ﻿SRS2220463. All RNA-Seq data has been deposited in the NCBI GEO database under accession GSE104198.

REFERENCES

Abyzov, A., A. E. Urban, M. Snyder and M. Gerstein, 2011 CNVnator: an approach to discover, genotype, and characterize typical and atypical CNVs from family and population genome sequencing. Genome Res 21**:** 974-984.

Anders, S., and W. Huber, 2010 Differential expression analysis for sequence count data. Genome Biology 11**:** R106.

Anders, S., P. T. Pyl and W. Huber, 2015 HTSeq--a Python framework to work with high-throughput sequencing data. Bioinformatics 31**:** 166-169.

Cingolani, P., A. Platts, L. Wang le, M. Coon, T. Nguyen *et al.*, 2012 A program for annotating and predicting the effects of single nucleotide polymorphisms, SnpEff: SNPs in the genome of Drosophila melanogaster strain w1118; iso-2; iso-3. Fly (Austin) 6**:** 80-92.

Faust, G. G., and I. M. Hall, 2014 SAMBLASTER: fast duplicate marking and structural variant read extraction. Bioinformatics 30**:** 2503-2505.

Garrison, E., and G. Marth, 2012 Haplotype-based variant detection from short-read sequencing. arXiv preprint arXiv:1207.3907.

Gish, S. R., E. J. Maier, B. C. Haynes, F. H. Santiago-Tirado, D. L. Srikanta *et al.*, 2016 Computational Analysis Reveals a Key Regulator of Cryptococcal Virulence and Determinant of Host Response. MBio 7.

Langmead, B., and S. L. Salzberg, 2012 Fast gapped-read alignment with Bowtie 2. Nat Methods 9**:** 357-359.

Langmead, B., C. Trapnell, M. Pop and S. L. Salzberg, 2009 Ultrafast and memory-efficient alignment of short DNA sequences to the human genome. Genome Biology 10**:** R25.

Layer, R. M., C. Chiang, A. R. Quinlan and I. M. Hall, 2014 LUMPY: a probabilistic framework for structural variant discovery. Genome Biol 15**:** R84.

Li, H., 2013 Aligning sequence reads, clone sequences and assembly contigs with BWAMEM. arXiv preprint arXiv:1303.3997v2.

Li, H., B. Handsaker, A. Wysoker, T. Fennell, J. Ruan *et al.*, 2009 The Sequence Alignment/Map format and SAMtools. Bioinformatics 25**:** 2078-2079.

Maier, E. J., B. C. Haynes, S. R. Gish, Z. A. Wang, M. L. Skowyra *et al.*, 2015 Model-driven mapping of transcriptional networks reveals the circuitry and dynamics of virulence regulation. Genome Res 25**:** 690-700.

Smit, A., R. Hubley and P. Green, 2013 RepeatMasker Open-4.0, pp.

Toffaletti, D. L., T. H. Rude, S. A. Johnston, D. T. Durack and J. R. Perfect, 1993 Gene transfer in *Cryptococcus neoformans* by use of biolistic delivery of DNA. J Bacteriol 175**:** 1405-1411.

Trapnell, C., L. Pachter and S. L. Salzberg, 2009 TopHat: discovering splice junctions with RNA-Seq. Bioinformatics 25**:** 1105-1111.
